# Supplementary material for: Urban soils along the Kern River and Los Gatos Creek are hotspots for Coccidioides in the San Joaquin Valley of California
Source: Appl Environ Microbiol. 2026 Sep 10;92(10):e00574-26. doi: 10.1128/aem.00574-26 (PMC13689388; doi:10.1128/aem.00574-26)
Supplement: Supplementary file 1 [file aem.00574-26-s0003.pdf]

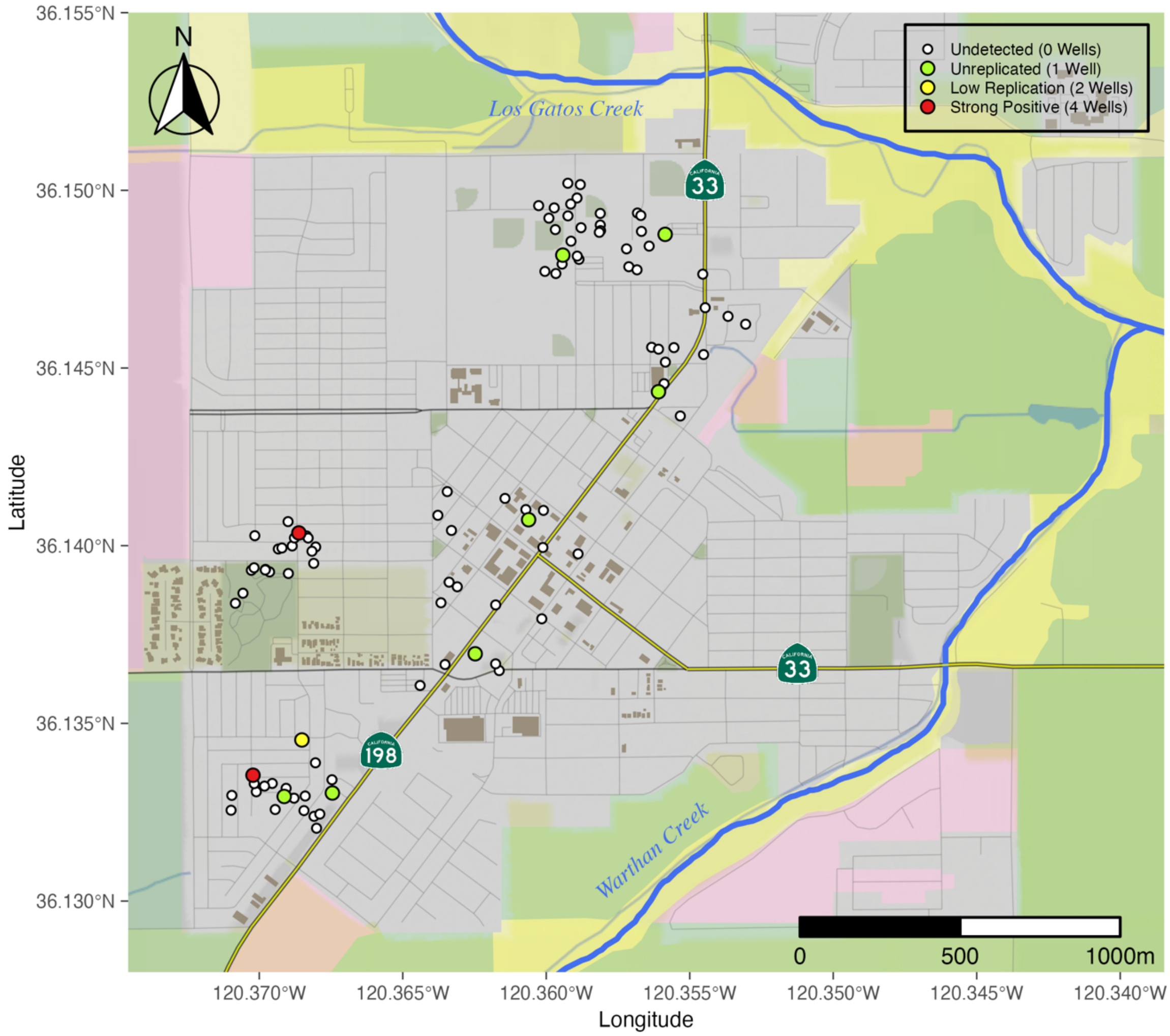

S1 Fig. Map of urban surfaces sampled in Coalinga, CA in August 2020 and tested using the CocciENV qPCR assay. n = 100. Point colors correspond to positive replicate wells. Map data (lines) were acquired from OpenStreetMap (www.openstreetmap.org) under the Open Data Commons Open Database License (OdbL) (https://www.openstreetmap.org/copyright). Map tiles are by Stamen Design (www.stamen.com) under Creative Commons By Attribution (CC BY 4.0) (https://creativecommons.org/licenses/by/4.0/).

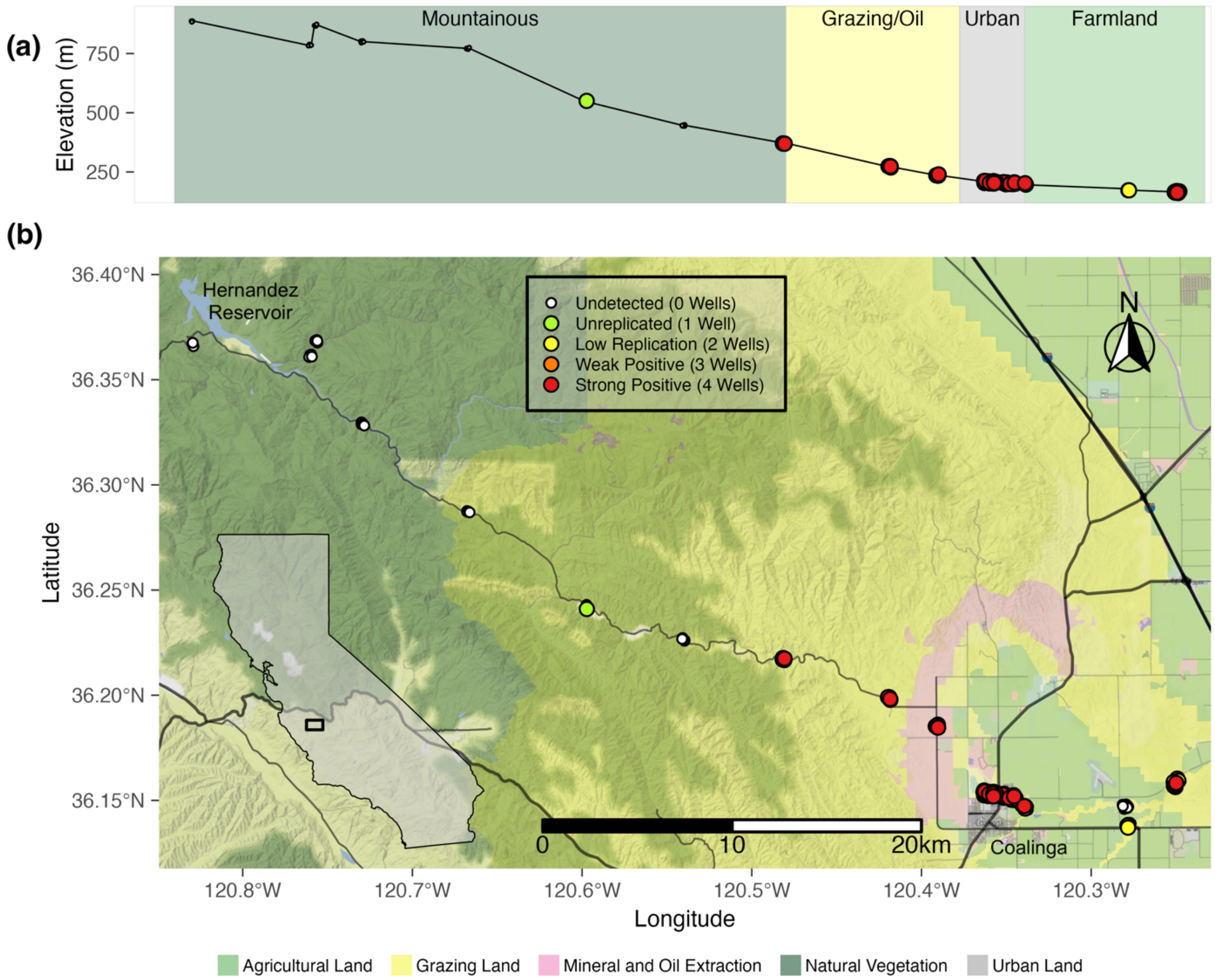

S2 Fig. Extended map of Los Gatos Creek rodent burrow soil sampling including April 2024 sites. All samples were tested using the CocciENV qPCR assay. Elevation profile (meters above sea level) and qualitative categorization of sample locations (a). Map of sample coordinates (b). Inset shows map extent. Landscape classification derived from the California Farmland Mapping and Monitoring Program 2018. Point colors correspond to positive replicate wells. Map data (lines) were acquired from OpenStreetMap (www.openstreetmap.org) under the Open Data Commons Open Database License (OdbL) (https://www.openstreetmap.org/copyright). Map tiles are by Stamen Design (www.stamen.com) under Creative Commons By Attribution (CC BY 4.0) (https://creativecommons.org/licenses/by/4.0/). California shapefile is public domain data by Natural Earth (www.naturalearthdata.com).

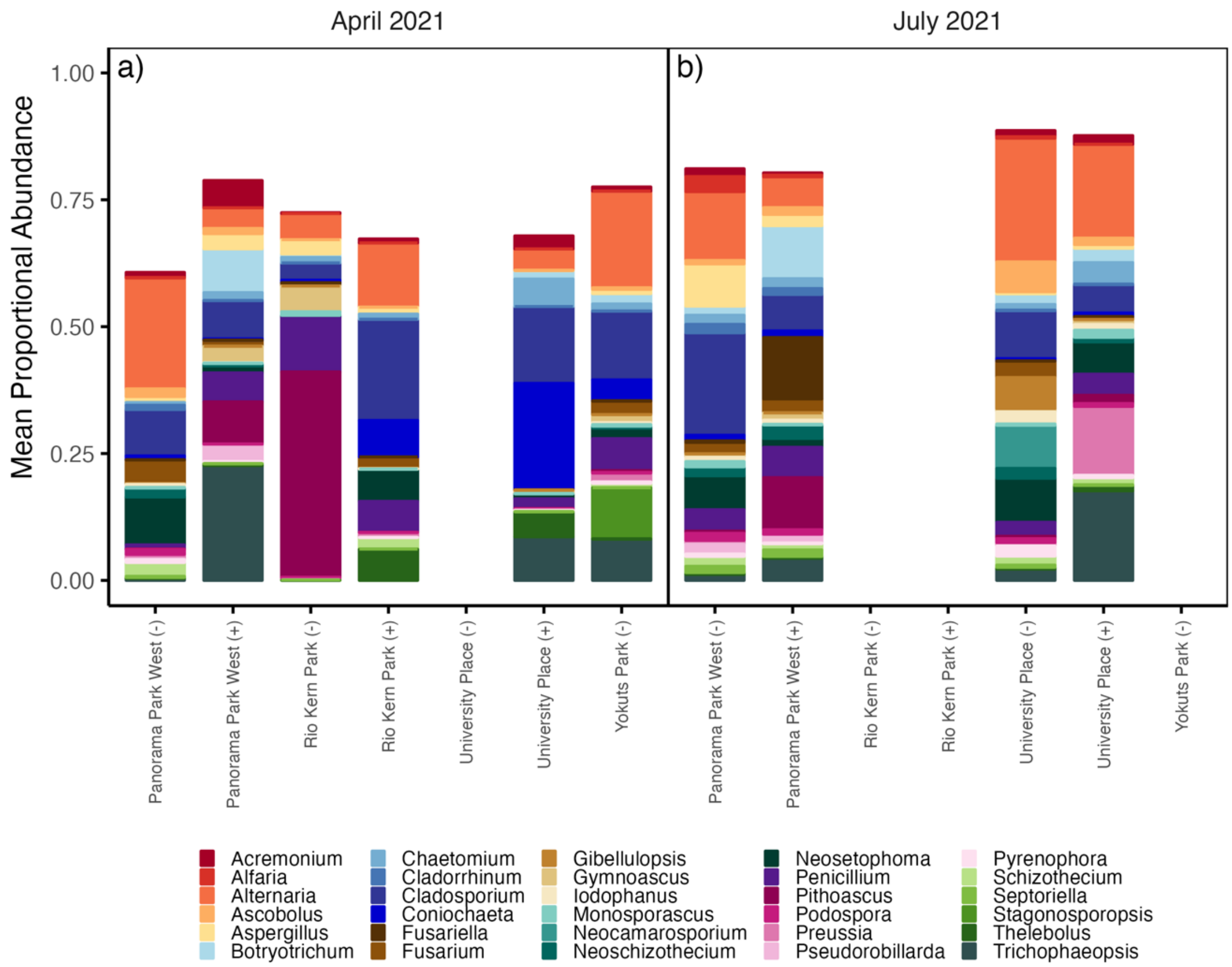

S3 Fig. The mean proportional abundance of the 30 most common fungal genera from sites along the Kern River as a function of site and sampling timepoint. (-) = *Coccidioides* negative samples. (+) = *Coccidioides* positive samples. n = 28.

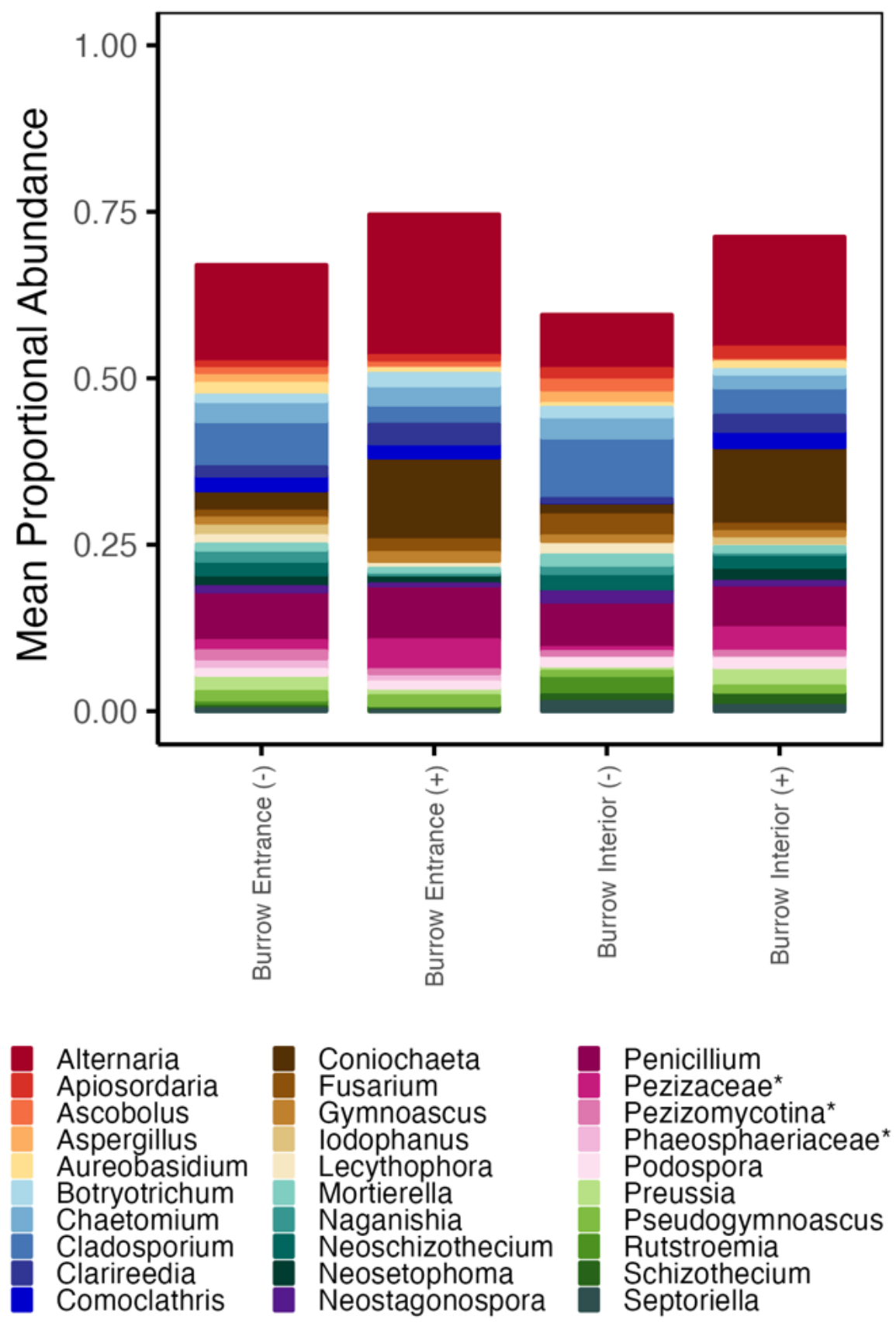

S4 Fig. The mean proportional abundance of the 30 most common fungal genera from sites along Los Gatos Creek as a function of sampling location (rodent burrow entrance vs interior). (-) = *Coccidioides* negative samples. (+) = *Coccidioides* positive samples. n = 40.

S1 Table. Sampling enumeration for the current study (**bold text**) and all other studies known to the authors where environmental *Coccidioides* was detected in soil, air, or both. The location and the method of detection are shown here. Total studies (including the current study) = 42. (+) = positive samples.

	Collection Location	Detection Method	Total	(+) Total	Burrow	(+) Burrow	non-Burrow	(+) non-Burrow	Airborne	(+) Airborne
**Current Study**	**California**	**CocciENV qPCR**	**1178**	**117**	**1058**	**107**	**20**	**10**	**100**	**2**
Detection of Airborne Coccidioides Spores Using Lightweight Portable Air Samplers Affixed to Uncrewed Aircraft Systems in California's Central Valley (Radosevich et. al 2025)	California	CocciENV qPCR	209	47	84	27	84	18	41	2
Understanding the exposure risk of aerosolized Coccidioides in a Valley fever endemic metropolis (Porter et. al 2024)	Arizona	CocciENV qPCR	5243	375	0	0	0	0	5243	375
Small mammals and their burrows shape the distribution of Coccidioides in soils: a long-term ecological experiment." (Head et. al 2024)	California	CocciENV qPCR	1988	322	1195	306	793	16	0	0
Análisis de la distribución actual de Coccidioides spp. en muestras de suelo de Baja California, México (Mota 2024)	Mexico	Nested PCR, ddPCR	76	5-30	76	5-30	0	0	0	0
Coccidioides undetected in soils from agricultural land and uncorrelated with time or the greater soil fungal community on undeveloped land (Wagner et. al 2023)	California	CocciENV qPCR	710	90	238	89	472	0	265	1
Risk of Exposure to Coccidioides spp. in the Temblor Special Recreation Management Area (SRMA), Kern County, CA (Lauer et. al 2023)	California	Nested PCR	114	36	15	5	99	31	0	0
Locking Down the Dust: Exploring the Association Between Coccidioides posadasii and Biological Soil Crusts (Ramsey 2023)	Arizona	CocciDX qPCR	80	2	20	2	60	0	0	0
Coccidioidomycosis in Northern Arizona: an Investigation of the Host, Pathogen, and Environment Using a Disease Triangle Approach (Mead et. al 2022)	Arizona	CocciDX/ ENV qPCR	171	6-26	171	6-26	0	0	0	0
The detection of Coccidioides from ambient air in Phoenix, Arizona: Evidence of uneven distribution and seasonality (Gade et. al 2020)	Arizona	Single Tube Nested qPCR	1009	0	0	0	0	0	1009	96-135
Valley Fever: Environmental Risk Factors and Exposure Pathways Deduced from Field Measurements in California (Lauer et. al 2020)	California	Nested PCR	389	122	0	0	389	122	0	0
Investigating the Role of Animal Burrows on the Ecology and Distribution of Coccidioides spp. in Arizona Soils (Kollath et. al 2020)	Arizona	CocciDX/ ENV qPCR	465	105	385	95	77	10	0	0
Earthquake-ridden area in USA contains Coccidioides, the Valley fever pathogen (Lauer et. al 2020)	California	Nested PCR	14	1	3	1	11	0	0	0
Direct detection of Coccidioides from Arizona soils using CocciENV, a highly sensitive and specific real-time PCR assay (Bowers et. al 2019)	Arizona	CocciENV qPCR	76	4	48	4	30	0	0	0
Detection of Coccidioides posadasii from xerophytic environments in Venezuela reveals risk of naturally acquired coccidioidomycosis infections (Alvarado 2018)	Venezuela	GeneSTAT.MDx qPCR	15	3-15	-	-	-	-	0	0
Large-scale land development, fugitive dust, and increased coccidioidomycosis incidence in the Antelope Valley of California, 1999—2014 (Colson et. al 2017)	California	Nested PCR	43	17	-	-	-	-	0	0
Molecular detection of airborne Coccidioides in Tucson, Arizona (Chow et. al 2016)	Arizona	Single Tube Nested qPCR	59	10	-	2	-	8	25	3
Impact of seasonal changes on fungal diversity of a semi-arid ecosystem revealed by 454 pyrosequencing (Vargas-Gastélum et. al 2015)	Mexico	Amplicon Sequencing	40	11	20	9	20	2	0	0
Valley Fever: Finding New Places for an Old Disease: Coccidioides immitis Found in Washington State Soil Associated With Recent Human Infection (Litvintseva 2015)	Washington	CocciDX qPCR, Culture	25	16	-	-	-	-	0	0
Demonstration of Coccidioides immitis and Coccidioides posadasii DNA in soil samples collected from Dinosaur National Monument, Utah (Johnson et. al 2014)	Utah	Nested PCR	2	2	1	1	1	1	0	0
Combining Forces - The Use of Landsat TM Satellite Imagery, Soil Parameter Information, and Multiplex PCR to Detect	California	Multiplex PCR	~192	12-144	-	-	-	-	-	-

Coccidioides immitis Growth Sites in Kern County, California (Lauer et. al 2014)										
Coccidioides immitis identified in soil outside of its known range - Washington, 2013 (Marsden-Haug et. al 2013)	Washington	CocciDX qPCR	22	6	0	0	0	0	0	0
Molecular detection of Coccidioides spp. from environmental samples in Baja California: linking Valley Fever to soil and climate conditions (Baptista-Rosas et. al 2012)	Mexico	Nested PCR	90	32	78	29	12	3	0	0
Detection and phylogenetic analysis of Coccidioides posadasii in Arizona soil samples (Barker et. al 2012)	Arizona	PCR, Mouse, Culture	700	11	-	5	-	6	0	0
Detection of Coccidioides immitis in Kern County, California, by multiplex PCR (Lauer et. al 2011)	California	PCR	546	31	0	0	546	31	0	0
Molecular identification of Coccidioides spp. in soil samples from Brazil (Macêdo et. al 2011)	Brazil	Nested PCR, Mouse Injection	24	6	24	6	0	0	0	0
Genomic and Population Analyses of the Mating Type Loci in Coccidioides Species Reveal Evidence for Sexual Reproduction and Gene Acquisition (Mandel et. al 2007)	Arizona	Mouse Injection	11	11	-	-	-	-	-	-
Phenotypic characterization and ecological features of Coccidioides spp. from Northeast Brazil (Cordeiro et. al 2006)	Brazil	Culture	14	3	14	3	0	0	0	0
Development of a Quantitative TaqMan™-PCR Assay and Feasibility of Atmospheric Collection for Coccidioides immitis for Ecological Studies (Daniels et. al 2002)	California	PCR	0	0	0	0	0	0	12	4
Outbreak of Coccidioidomycosis in Washington State Residents Returning from Mexico (Cairns et. al 2000)	Mexico	Mouse Injection	3	3	-	-	-	-	-	-
Soil isolation and molecular identification of Coccidioides immitis (Greene et. al 2000)	California	PCR	720	4	0	0	720	4	0	0
Soil Ecology of Coccidioides immitis at Amerindian Middens in California (Lacy and Swatek 1974)	California	Culture	325	32	0	0	325	32	0	0
Some fungi isolated with Coccidioides immitis from soils of endemic areas in California (Orr 1968)	California	Culture	22	22	0	0	22	22	0	0
Recovery of Coccidioides immitis from the air (Ajello et. al 1965)	Arizona	Mouse Injection, Culture	0	0	0	0	0	0	128	2
Observations on Coccidioides immitis found growing naturally in soil. (Maddy et. al 1965)	Arizona	Culture, Injection	198	30	0	0	198	30	0	0
Isolation of Coccidioides immitis from Soil (Levine et. al 1964)	California	Culture, Mouse Injection	37	4	-	-	-	-	0	0
Growth Patterns of Coccidioides immitis (Elconin 1957)	California	Culture, Mouse	428	31	115	18	313	13	0	0
Ecological studies of Coccidioides immitis (Plunkett and Swatek 1957)	California	Culture	80	0	80	0	0	0	0	0
Coccidioides immitis in the soil of the southern San Joaquin Valley. (Egeberg and Ely 1954)	California	Culture, Injection	500	35	177	24	323	11	0	0
Isolation of Coccidioides from soil and rodents (Emmons 1942)	Arizona	Isolation	150	5	-	-	-	-	0	0
An Epidemic Of Coccidioidal Infection (Coccidioidomycosis) (Davis et. al 1942)	California	Isolation	1	1	1	0	0	0	0	0
Isolation of Coccidioides Immitis (Stiles) from the Soil (Stewart and Meyer 1932)	California	Culture, Guinea Pig	>1	>1	-	-	-	-	0	0

S2 Table. *Coccidioides* detection in soils and settled dust using the CocciEnv qPCR assay as a function of site and detection level. Numbers in parentheses indicate the minimum proportion of replicate wells with CT < 40 and logarithmic amplification. Only samples detected in ≥ 3 replicate wells were considered positive detections. * = Bakersfield area / non-Kern River site

	Undetected (0/4)	Unreplicated (1/4)	Low Replication (2/4)	Weak Positive (3/4)	Strong Positive (4/4)	Total
**Kern River (soils)**						
1. Buena Vista Valley * (non-Kern River site)	13	2	0	0	0	15
2. California Aqueduct * (non-Kern River site)	8	4	0	0	0	12
3. Buena Vista Recreation Area * (non-Kern River	45	1	0	0	0	46
4. Kern River Mouth	1	1	0	0	0	2
5. Kern River Parkway West End	59	1	1	1	1	63
6. Highgate Park	3	0	0	0	0	3
7. River Walk	4	2	0	0	0	6
8. CSUB West * (non-Kern River site)	38	0	0	0	0	38
9. CSUB North * (non-Kern River site)	7	0	0	0	0	7
10. University Place	68	7	6	4	21	106
11. Sports Village West * (non-Kern River site)	20	0	0	0	0	20
12. Sports Village East * (non-Kern River site)	65	2	0	0	0	67
13. Rio Kern Park	55	5	0	2	0	62
14. Truxtun Park	20	0	0	0	0	20
15. Yokuts Park	60	1	2	0	0	63
16. Beach Park	26	0	0	0	0	26
17. Upland Park	3	0	0	0	0	3
18. Panorama Vista Preserve	3	0	0	0	0	3
19. Panorama Park West	75	0	1	0	20	96
20. Greenlawn Cemetery	6	0	0	0	0	6
21. Panorama Park East	17	1	0	0	2	20
22. Kern River Parkway East Bridge	2	1	0	0	0	3
23. Kern River Parkway East End	21	0	0	1	0	22
24. Gordons Ferry	1	2	0	0	0	3
25. Round Mountain Road	3	0	0	0	0	3
26. Hart Park West	3	0	0	0	0	3
27. Hart Park East	3	0	0	0	0	3
28. Kern County Soccer Park	29	0	0	0	0	29
29. Kern River Golf Course	4	0	0	0	0	4
30. Rancheria Road	6	0	0	0	0	6
31. Upper Richbar	6	0	0	0	0	6
32. Kern Canyon Road West End	6	0	0	0	0	6
33. Kern Canyon Road 4km	6	0	0	0	0	6
34. Kern Canyon Road 11km	6	0	0	0	0	6
35. Remington Ridge Trailhead	7	0	0	0	0	7
36. Hobo Campground	3	0	0	0	0	3
37. Sandy Flat Campground	7	0	0	0	0	7
38. Tank Park	6	0	0	0	0	6
39. Isabella Hot Springs	5	0	0	0	0	5
40. Auxiliary Dam Campground	6	0	0	0	0	6
41. Old Isabella	6	0	0	0	0	6
42. South Fork Rec Campground	6	0	0	0	0	6
	**738**	**30**	**10**	**8**	**44**	**830**
**Los Gatos Creek (soils)**						
W01. Laguna Mountain Campground	3	0	0	0	0	3
W02. Oak Flat Campground	4	0	0	0	0	4
W03. Jade Mill Campground	6	0	0	0	0	6
W04. Mile 4.3	5	0	0	0	0	5
W05. Mile 9.4	5	0	0	0	0	5
W06. Benitoite Mine	4	1	0	0	0	5
W07. Mile 19.5	5	0	0	0	0	5
W08. Mile 24	1	0	0	0	4	5
W09. Mile 28.6	0	0	0	0	5	5
W10. Mile 31.1	0	0	0	3	2	5
1A. Highway 33 West (burrows)	34	3	6	4	13	60
1B. Highway 33 West (surface soils)	8	0	2	4	6	20
2. Highway 33 East	24	0	1	1	14	40
3. Warthan Creek	18	0	0	1	1	20
4. South Calavaras	20	0	0	0	0	20
5. Jacalitos Creek	18	1	1	0	0	20
6. Phelps Avenue 10km	11	2	2	0	5	20
	**166**	**7**	**12**	**13**	**50**	**248**
**Coalinga (urban surfaces)**						
1. Coalinga	90	7	1	0	2	100
	**90**	**7**	**1**	**0**	**2**	**100**
**Total**	994	44	23	21	96	1178

S3 Table. *Coccidioides* detection using the CocciEnv qPCR assay from sites with >1 timepoint as a function of site, date and detection level. Numbers in parentheses indicate the minimum proportion of replicate wells with CT < 40 and logarithmic amplification. * = Bakersfield area / non-Kern River site

		Undetected (0/4)	Unreplicated (1/4)	Low Replication (2/4)	Weak Positive (3/4)	Strong Positive (4/4)	Total
**Kern River (soils)**							
3. Buena Vista Recreation Area * (non-Kern River site)	Apr 2021	6	0	0	0	0	6
	Jan 2022	39	1	0	0	0	40
5. Kern River Parkway West End	Apr 2021	2	1	0	0	0	3
	Jul 2021	20	0	0	0	0	20
	Oct 2021	20	0	0	0	0	20
	Jan 2022	17	0	1	1	1	20
8. CSUB West * (non-Kern River site)	Jul 2021	20	0	0	0	0	20
	Oct 2021	18	0	0	0	0	18
9. CSUB North * (non-Kern River site)	Jul 2021	4	0	0	0	0	4
	Oct 2021	3	0	0	0	0	3
10. University Place	Apr 2021	1	4	0	1	0	6
	Jul 2021	14	1	1	0	4	20
	Oct 2021	16	0	0	0	4	20
	Jan 2022	37	2	5	3	13	60
12. Sports Village East * (non-Kern River site)	Apr 2021	24	2	0	0	0	26
	Jul 2021	21	0	0	0	0	21
	Oct 2021	20	0	0	0	0	20
13. Rio Kern Park	Apr 2021	1	1	0	1	0	3
	Jul 2021	19	1	0	0	0	20
	Oct 2021	18	1	0	0	0	19
	Jan 2022	17	2	0	1	0	20
15. Yokuts Park	Apr 2021	2	0	1	0	0	3
	Jul 2021	19	1	0	0	0	20
	Oct 2021	19	0	1	0	0	20
	Jan 2022	20	0	0	0	0	20
16. Beach Park	Apr 2021	6	0	0	0	0	6
	Jan 2022	20	0	0	0	0	20
19. Panorama Park West	Apr 2021	4	0	0	0	2	6
	Jul 2021	15	0	1	0	4	20
	Oct 2021	26	0	0	0	4	30
	Jan 2022	30	0	0	0	10	40
28. Kern County Soccer Park	Apr 2021	9	0	0	0	0	9
	Oct 2021	20	0	0	0	0	20
		**527**	**17**	**10**	**7**	**42**	**603**
**Los Gatos Creek (soils)**							
1A. Highway 33 West (burrows)	Aug 2020	6	2	1	3	8	20
	Jan 2022	28	1	5	1	5	40
1B. Highway 33 West (surface soils)	Aug 2020	8	0	2	4	6	20
		**42**	**3**	**8**	**8**	**19**	**80**
**Total**		**569**	**20**	**18**	**15**	**61**	**683**

S4 Table. Enumeration of subset of soil samples selected for ITS2 amplicon sequencing. n = 35 negative samples; 33 positive samples.

Waterway	Burrow position	Timepoint	Site	*Coccidioides*	# of Samples
Los Gatos Creek	Burrow Entrance	Aug 2020	Highway 33 West	Negative (-)	10
Los Gatos Creek	Burrow Interior	Aug 2020	Highway 33 West	Negative (-)	9
Kern River	Burrow Interior	Apr 2021	Panorama Park West	Negative (-)	2
Kern River	Burrow Interior	Jul 2021	Panorama Park West	Negative (-)	6
Kern River	Burrow Interior	Apr 2021	Rio Kern Park	Negative (-)	1
Kern River	Burrow Interior	Jul 2021	University Place	Negative (-)	4
Kern River	Burrow Interior	Apr 2021	Yokuts Park	Negative (-)	3
Los Gatos Creek	Burrow Entrance	Aug 2020	Highway 33 West	Positive (+)	10
Los Gatos Creek	Burrow Interior	Aug 2020	Highway 33 West	Positive (+)	11
Kern River	Burrow Interior	Apr 2021	Panorama Park West	Positive (+)	2
Kern River	Burrow Interior	Jul 2021	Panorama Park West	Positive (+)	4
Kern River	Burrow Interior	Apr 2021	Rio Kern Park	Positive (+)	1
Kern River	Burrow Interior	Apr 2021	University Place	Positive (+)	1
Kern River	Burrow Interior	Jul 2021	University Place	Positive (+)	4
**Total**					**68**

S5 Table. Generalized Linear Mixed Model (GLMM) coefficient table showing *Coccidioides* detection as a function of elevation, sampling month and soil moisture data (fixed effects), with site included as a random effect, across all Kern River sites. n = 830.

	Estimate	OR	CI 2.5%	CI 97.5%	SE	z-value	p-value	
Intercept	-6.926	9.82E-04	3.36E-05	0.03	1.721	-4.024	0	***
Elevation	4.463	86.75	0.62	1.21E+04	2.518	1.773	0.076	.
Surface Soil Moisture (0-5cm)	-2.736	0.06	4.25E-03	0.99	1.39	-1.968	0.049	*
Rootzone Soil Moisture (0-100cm)	-4.786	8.35E-03	2.61E-05	2.67	2.943	-1.626	0.104	
2021 July	-4.028	0.02	3.42E-04	0.93	2.017	-1.996	0.046	*
2021 October	-3.817	0.02	5.89E-04	0.82	1.847	-2.066	0.039	*
2022 January	7.834	2.52E+03	1.40	4.54E+06	3.824	2.049	0.04	*

. = $p < 0.1$, * = $p < 0.05$, ** = $p < 0.01$, *** = $p \leq 0.001$, OR = Odds Ratio, CI = Confidence Interval, SE = Standard Error

S6 Table. Modified generalized Linear Mixed Model (GLMM) coefficient table, excluding samples collected above 210 meters showing *Coccidioides* detection as a function of elevation, sampling month and soil moisture data (fixed effects), with site included as a random effect, across all Kern River sites (below 210 meters). n = 760.

	Estimate	OR	CI 2.5%	CI 97.5%	SE	z-value	p-value	
Intercept	-13.844	4.96E-03	2.09E-04	0.12	4.204	-3.293	0.001	***
Elevation	0.048	3.70E+03	5.26	2.61E+06	0.02	2.456	0.014	*
Surface Soil Moisture (0-5cm)	-2.272	0.10	7.16E-03	1.48	1.361	-1.67	0.095	.
Rootzone Soil Moisture (0-100cm)	-4.287	0.01	7.65E-05	2.47	2.649	-1.619	0.106	
2021 July	-3.594	0.03	6.82E-04	1.11	1.887	-1.905	0.057	.
2021 October	-3.343	0.04	1.21E-03	1.03	1.721	-1.942	0.052	.
2022 January	6.869	961.62	0.88	1.06E+06	3.573	1.923	0.055	.

. = $p < 0.1$, * = $p < 0.05$, ** = $p < 0.01$, *** = $p \leq 0.001$, OR = Odds Ratio, CI = Confidence Interval, SE = Standard Error

S7 Table. Logistic regression coefficient table showing *Coccidioides* detection as a function of sampling site, sampling month and soil moisture data between Kern River sites at Panorama Park West and University Place. n = 202.

	Estimate	OR	CI 2.5%	CI 97.5%	SE	z-value	p-value
Intercept	-4.878	0.01	5.35E-24	3.82E+18	24.209	-0.201	0.84
July 2021	0.899	2.46	0.00	4.88E+06	7.21	0.125	0.90
October 2021	0.587	1.80	0.00	1.34E+06	6.673	0.088	0.93
January 2022	-2.055	0.13	1.91E-13	7.46E+10	13.647	-0.151	0.88
Site (Panorama Park West)	-1.105	0.33	0.00	1.99E+04	5.571	-0.198	0.84
Surface Soil Moisture (0-5cm)	12.703	3.29E+05	5.62E-104	7.20E+115	127.011	0.1	0.92
Rootzone Soil Moisture (0-100cm)	44.459	2.03E+19	1.63E-193	1.08E+235	247.267	0.18	0.86

. = $p < 0.1$, * = $p < 0.05$, ** = $p < 0.01$, *** = $p \leq 0.001$, OR = Odds Ratio, CI = Confidence Interval, SE = Standard Error

S8 Table. PERMANOVA coefficient table showing the association between the soil fungal community and *Coccidioides* detection (Presence), as a function of sampling site and sampling date, from rodent burrows soils collected from along the Kern River. *Coccidioides* detection determined using the CocciEnv qPCR assay. Fungal community structure modeled as species-level Bray-Curtis dissimilarities. Permutations = 1000. n = 28.

	Degrees of Freedom	Sum of Squares	r^2	Pseudo-F	p-value	
Coccidioides Presence	1	0.327	0.03	1.067	0.395	
Site	3	2.145	0.22	2.335	0.002	**
Date	1	0.394	0.04	1.286	0.234	
Coccidioides Presence : Site	2	0.83	0.09	1.355	0.145	
Coccidioides Presence : Date	1	0.217	0.02	0.71	0.702	
Site : Date	1	0.382	0.04	1.247	0.253	
Residual	18	5.512	0.56			
**Total**	**27**	**9.807**	**1**			

. = $p < 0.1$, * = $p < 0.05$, ** = $p < 0.01$, *** = $p \leq 0.001$

S9 Table. Pairwise fungal community differences (PERMANOVA) between Kern River sites using the ‘pairwiseadonis2’ function. Number of samples collected at each site shown in parentheses. Permutations = 1000. n = 28.

Contrast	Degrees of Freedom	Sum of Squares	r^2	Pseudo-F	p-value	
Panorama Park West (14) : University Place (9)	1	0.7	0.08	1.72	0.04	*
Panorama Park West (14) : Rio Kern Park (2)	1	0.35	0.06	0.82	0.71	
Panorama Park West (14) : Yokuts Park (3)	1	0.42	0.06	0.99	0.42	
University Place (9) : Rio Kern Park (2)	1	0.23	0.06	0.58	0.93	
University Place (9) : Yokuts Park (3)	1	0.43	0.10	1.09	0.32	
Rio Kern Park (2) : Yokuts Park (3)	1	0.42	0.25	0.98	0.40	

. = $p < 0.1$, * = $p < 0.05$, ** = $p < 0.01$, *** = $p \leq 0.001$

S10 Table. PERMANOVA coefficient table showing the association between the soil fungal community, *Coccidioides* detection (Presence) and sampling location (burrow interior vs entrance) from rodent burrow soils collected in August 2020 from the Los Gatos Creek "Hwy33 West" site. *Coccidioides* detection determined using the CocciEnv qPCR assay. Fungal community structure modeled as species-level Bray-Curtis dissimilarities. Permutations = 1000. n = 40.

	Degrees of Freedom	Sum of Squares	r^2	Pseudo-F	p-value
Coccidioides Presence	1	0.373	0.03	1.174	0.234
Sampling Location	1	0.318	0.03	1	0.381
Coccidioides Presence : Sampling Location	1	0.269	0.02	0.846	0.682
Residual	36	11.435	0.92		
**Total**	**39**	**12.394**	**1**		

. = p < 0.1, * = p < 0.05, ** = p < 0.01, *** = p ≤ 0.001

S11 Table. Percent abundance of the 30 most abundant fungal genera at Kern River and Los Gatos Creek Sites. Values are means between replicates. Underlined genera appear uniquely in the top 30 genera at each location.

Kern River			Los Gatos Creek		
	mean	sem		mean	sem
Alternaria	14.58	2.05	Alternaria	15.38	1.67
Cladosporium	11.51	1.61	Coniochaeta	7.6	1.81
Trichophaeopsis	6.53	2.6	Penicillium	7.35	1.61
Neosetophoma	5.09	1.31	Cladosporium	5.78	0.91
Penicillium	4.56	0.81	Chaetomium	2.77	0.64
Pithoascus	3.94	1.78	Pezizaceae*	2.61	0.6
Botryotrichum	3.52	1.05	Preussia	2.03	0.61
Aspergillus	2.79	1.61	Comoclathris	1.78	0.61
Preussia	2.6	1.33	Fusarium	1.75	0.33
Fusarium	2.22	0.42	Clarireedia	1.69	0.75
Coniochaeta	2.12	0.82	Neosetophoma	1.65	0.5
Chaetomium	1.99	0.4	Neoschizothecium	1.58	0.48
Fusariella	1.81	1.72	Iodophanus	1.18	0.34
Ascobolus	1.58	0.47	Aspergillus	1.16	0.42
Acremonium	1.47	0.4	Phaeosphaeriaceae*	1.14	0.24
Schizothecium	1.44	0.32	Ascobolus	1.1	0.29
Neocamarosporium	1.3	1.03	Rutstroemia	1.1	0.41
Neoschizothecium	1.28	0.34	Aureobasidium	1.08	0.19
Gymnoascus	1.19	0.25	Schizothecium	1.03	0.3
Stagonosporopsis	1.14	1.07	Botryotrichum	1.01	0.21
Gibellulopsis	0.89	0.84	Lecythophora	0.96	0.49
Iodophanus	0.89	0.32	Naganishia	0.9	0.26
Pseudorobillarda	0.83	0.41	Pseudogymnoascus	0.9	0.34
Alfaria	0.73	0.63	Podospora	0.9	0.16
Thelebolus	0.72	0.29	Apiosordaria	0.83	0.3
Podospora	0.7	0.2	Mortierella	0.75	0.14
Cladorrhinum	0.62	0.28	Gymnoascus	0.69	0.16
Pyrenophora	0.57	0.26	Septoriella	0.57	0.28
Monosporascus	0.56	0.26	Neostagonospora	0.55	0.26
Septoriella	0.55	0.19	Pezizomycotina*	0.55	0.15

* = Incertae sedis, sem = standard error of the mean

S12 Table: Indicator species for *Coccidioides* positive and *Coccidioides* negative soil samples collected from within Kern River rodent burrows. Significance was calculated in indicspecies version 1.7.12 with 1000 permutations. Sequences were cross-referenced via NCBI Nucleotide Blast (https://blast.ncbi.nlm.nih.gov/) for each prospective indicator species. • = equally likely alternative species (in subsection). IndVal and p-value apply to all alternative species.

Coccidioides positive samples (n = 12)

	BLASTN %	Reads	IndVal	p-value	
Aspergillus salinicola	99.2	3044	0.67	0.018	*
Gymnoascus dugwayensis	98.5	8128	0.849	0.012	*
Coniochaeta discospora	100.0	6449	0.909	0.008	**
Massariosphaeria roumeguerei	95.1	516	0.69	0.025	*
Montagnea arenaria	99.5	780	0.816	0.001	***
Myxotrichum deflexum	100.0	3065	0.847	0.023	*
Penicillium citreonigrum	100.0	847	0.56	0.022	*
• *Penicillium restrictum*					
• *Penicillium fundyense*					
• *Penicillium toxicarium*					
Pleuroascus nicholsonii	100.0	2244	0.867	0.01	*

Coccidioides negative samples (n = 16)

	BLASTN %	Reads	IndVal	p-value	
Cladorrhinum flexuosum	99.7	2969	0.773	0.037	*
Fusariella hughesii	99.1	1182	0.783	0.022	*
Fusarium solani	100.0	969	0.658	0.031	*
• *Fusarium falciforme*					
• *Fusarium obliquiseptatum*					
• *Umbelopsis nana*					
• *Fusarium oxysporum*					

* = $p < 0.05$, ** = $p < 0.01$, *** = $p \leq 0.001$, IndVal = indicator value

S13 Table: Indicator species for *Coccidioides* positive soil samples collected from within Los Gato Creek rodent burrows. Significance was calculated in indicspecies version 1.7.12 with 1000 permutations. Sequences were cross-referenced via NCBI Nucleotide Blast (https://blast.ncbi.nlm.nih.gov/) for each prospective indicator species. • = equally likely alternative species (in subsection). IndVal and p-value apply to all alternative species.

	Coccidioides positive samples (n = 11)				
	BLASTN %	Reads	IndVal	p-value	
Alternaria lolii	100.0	4095	0.774	0.012	*
Coniochaeta polymorpha	98.4	15515	0.897	0.006	**
Kalmusia utahensis	100.0	151	0.739	0.019	*
Monosporascus ibericus	96.8	577	0.739	0.017	*
Preussia polymorpha	98.1	6910	0.888	0.048	*
Sporormiella megalospora	98.1				
Preussia terricola	98.1				

* = p < 0.05, ** = p < 0.01, *** = p ≤ 0.001, IndVal = indicator value

S14 Table: Indicator species for *Coccidioides* negative soil samples collected from within Los Gatos Creek rodent burrows. Significance was calculated in indicspecies version 1.7.12 with 1000 permutations. Sequences were cross-referenced via NCBI Nucleotide Blast (https://blast.ncbi.nlm.nih.gov/) for each prospective indicator species. • = equally likely alternative species (in subsection). IndVal and p-value apply to all alternative species.

Coccidioides negative samples (n = 9)

		BLASTN %	Reads	IndVal	p-value	
Aspergillus cristatus<br>• *Aspergillus amstelodami*<br>• *Aspergillus chevalieri*<br>• *Aspergillus medius*<br>• *Aspergillus ruber*<br>• *Aspergillus costiformis*	• *Aspergillus intermedius*<br>• *Aspergillus montevidensis*<br>• *Aspergillus medius*<br>• *Aspergillus heterocaryoticus*<br>• *Aspergillus caperatus*	100.0	331	0.745	0.009	**
Chaetomium madrasense<br>• *Chaetomium contagiosum*<br>• *Chaetomium grande*<br>• *Chaetomium interruptum*	• *Chaetomium megalocarpum*<br>• *Chaetomium ascotrichoides*<br>• *Chaetomium globosum*	100.0	3542	0.79	0.007	**
Circinella muscae	• *Fennellomyces linderi*	100.0	949	0.739	0.033	*
Holtermanniella festucosa<br>• *Holtermanniella wattica*	• *Holtermanniella takashimae*<br>• *Holtermanniella festucosa*	99.4	482	0.899	0.003	**
Dactylonectria torresensis	• *Dactylonectria macrodidyma*	100.0	180	0.732	0.028	*
Enterocarpus grenotii	• *Basipetospora chlamydospora*	99.1	1795	0.822	0.014	*
Ustilago bullata		100.0	169	0.786	0.009	**
Fusarium oxysporum<br>• *Fusarium solani*<br>• *Fusarium foetens*	• *Fusarium graminearum*<br>• *Fusarium fujikuroi*	100.0	549	0.667	0.026	*
Mortierella alpina		100.0	577	0.808	0.013	*
Linnemannia gamsii		98.5	441	0.717	0.038	*
Myrmecridium schulzeri		100.0	680	0.751	0.046	*
Paraphoma fimeti	• *Septoriella neoarundinis*	100.0	365	0.803	0.004	**
Pseudoechria decidua		96.2	173	0.745	0.008	**
Solicoccozyma aeria		99.7	1189	0.814	0.011	*
Phoma sojicola<br>• *Ascochyta rabiei*<br>• *Nothophoma quercina*<br>• *Stagonosporopsis*	• *Microsphaeropsis olivacea*<br>• *Allophoma zantedeschiae*<br>• *Allophoma labilis*<br>• *Ascochyta medicaginicola*	99.3	219	0.667	0.03	*
Tetracladium furcatum	• *Tetracladium setigerum*	99.6	157	0.725	0.029	*

* = p < 0.05, ** = p < 0.01, *** = p ≤ 0.001, IndVal = indicator value

S15 Table: Indicator species for *Coccidioides* samples (positive and negative) collected from surface soils outside Los Gatos Creek rodent burrows. Significance was calculated in indicspecies version 1.7.12 with 1000 permutations. Sequences were cross-referenced via NCBI Nucleotide Blast (https://blast.ncbi.nlm.nih.gov/) for each prospective indicator species. • = equally likely alternative species (in subsection). IndVal and p-value apply to all alternative species.

Coccidioides positive samples (n = 10)

	BLASTN %	Reads	IndVal	p-value	
Coniosporium apollinis	100.0	4420	0.91	0.014	*
Coprinopsis gonophylla	99.7	2394	0.831	0.02	*
Mycotypha microspora	100.0	155	0.747	0.029	*

Coccidioides negative samples (n = 10)

	BLASTN %	Reads	IndVal	p-value	
Dothiora viticola	99.4	487	0.775	0.008	**
Podospora vesticola • *Schizothecium carpinicola*	99.0	3908	0.768	0.023	*
Ramimonilia apicalis	97.6	536	0.759	0.042	*

* = $p < 0.05$, ** = $p < 0.01$, *** = $p \leq 0.001$, IndVal = indicator value

S1 Data: All non-sequencing data and metadata used in this study, excluding remotely sensed soil moisture data.

S2 Data: All remotely sensed soil moisture data used in this study.

S1 Code: All code needed to process sequencing data and to generate taxonomic tables.

S2 Code: All code needed to replicate statistical analyses.
